# (1,4,7,10-Tetra­aza­cyclo­dodecane-κ^4^ *N* ^1^,*N* ^4^,*N* ^7^,*N* ^10^)(tetra­oxidomolybdato-κ*O*)copper(II) monohydrate

**DOI:** 10.1107/S1600536810026000

**Published:** 2010-07-07

**Authors:** Dorothea Rohde, Kurt Merzweiler

**Affiliations:** a Institut für Chemie, Naturwisssenchaftliche Fakultät II, Martin-Luther-Universität Halle-Wittenberg, Kurt-Mothes-Strasse 2, 06120 Halle, Germany

## Abstract

In the title compound, [CuMoO_4_(C_8_H_20_N_4_)]·H_2_O, the Cu^II^ atom is coordinated by four N atoms of the 1,4,7,10-tetra­aza­cyclo­dodecane (cyclen) ligand and one O atom of the molybdate unit in a distorted square-pyramidal environment. The water mol­ecules are linked to the complex unit to form centrosymmetric dimers [*R*_4_^4^(12) and *R*_4_^4^(16)] and discrete *D*_3_^2^(9), *D*_3_^3^(11) and *D*_3_^3^(13) chains by O—H⋯O and N—H⋯O inter­actions. Additionally, the complex mol­ecules are linked into *C*_4_^4^(18) chain motifs by N—H⋯O inter­actions. As a result [(cyclen)CuMoO_4_] units and water molecules are linked to  layers that are oriented parallel to the *ac* plane. The stacking of the layers in the *b*-axis direction is supported by weak C—H⋯O hydrogen bridges.

## Related literature

For inorganic–organic hybrid materials based on copper complexes with bridging molybdate ligands, see, for example: Rarig *et al.* (2002 ▶); Hagrman *et al.* (1998 ▶). For copper complexes with the cyclen ligand, see: Clay *et al.* (1979 ▶); Lu *et al.* (1997 ▶); Yeung *et al.* (2000 ▶); Guo *et al.* (2008 ▶). For related literature, see: Bernstein *et al.* (1995 ▶); Choi *et al.* (2004 ▶).
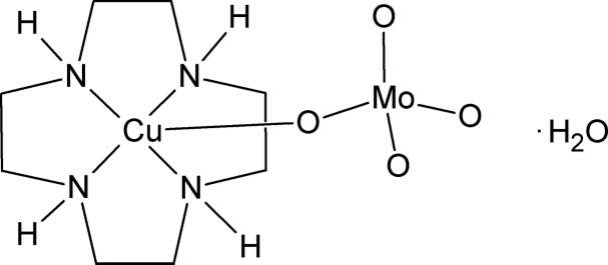


## Experimental

### 

#### Crystal data

[CuMoO_4_(C_8_H_20_N_4_)]·H_2_O
*M* *_r_* = 413.78
Triclinic, 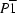

*a* = 8.6985 (6) Å
*b* = 8.9784 (6) Å
*c* = 9.0055 (6) Å
α = 90.358 (6)°
β = 91.949 (6)°
γ = 100.742 (5)°
*V* = 690.54 (8) Å^3^
*Z* = 2
Mo *K*α radiation
μ = 2.47 mm^−1^
*T* = 200 K
0.22 × 0.21 × 0.13 mm

#### Data collection

Stoe IPDS 2T diffractometer
Absorption correction: numerical (*X-RED*; Stoe & Cie, 2009 ▶) *T*_min_ = 0.612, *T*_max_ = 0.737
9700 measured reflections
3017 independent reflections
2788 reflections with *I* > 2σ(*I*)
*R*_int_ = 0.037

#### Refinement

*R*[*F* ^2^ > 2σ(*F* ^2^)] = 0.024
*wR*(*F* ^2^) = 0.063
*S* = 1.04
3017 reflections
196 parameters
6 restraints
H atoms treated by a mixture of independent and constrained refinement
Δρ_max_ = 0.48 e Å^−3^
Δρ_min_ = −1.05 e Å^−3^

### 

Data collection: *X-AREA* (Stoe & Cie, 2009 ▶); cell refinement: *X-AREA*; data reduction: *X-RED* (Stoe & Cie, 2009 ▶); program(s) used to solve structure: *SHELXS97* (Sheldrick, 2008 ▶); program(s) used to refine structure: *SHELXL97* (Sheldrick, 2008 ▶); molecular graphics: *DIAMOND* (Brandenburg, 2009 ▶); software used to prepare material for publication: *SHELXL97* and *PLATON* (Spek, 2009 ▶).

## Supplementary Material

Crystal structure: contains datablocks I, global. DOI: 10.1107/S1600536810026000/bx2287sup1.cif

Structure factors: contains datablocks I. DOI: 10.1107/S1600536810026000/bx2287Isup2.hkl

Additional supplementary materials:  crystallographic information; 3D view; checkCIF report

## Figures and Tables

**Table 1:** Hydrogen-bond geometry (Å, °)

*D*—H⋯*A*	*D*—H	H⋯*A*	*D*⋯*A*	*D*—H⋯*A*
O5—H5⋯O4^i^	0.83 (2)	1.95 (2)	2.770 (2)	169 (3)
N1—H1⋯O5^ii^	0.86 (2)	2.35 (2)	3.156 (3)	156 (3)
N2—H2⋯O1^ii^	0.89 (2)	2.19 (2)	2.949 (2)	143 (3)
N3—H3⋯O4^i^	0.88 (2)	2.28 (3)	2.955 (3)	133 (3)
N4—H4⋯O5^iii^	0.88 (2)	2.10 (2)	2.928 (3)	157 (3)
O5—H6⋯O2^iv^	0.84 (2)	1.85 (2)	2.666 (3)	163 (4)
C3—H3*B*⋯O3^v^	0.99	2.36	3.345 (3)	175

Symmetry codes: (i) 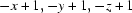; (ii) 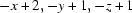; (iii) 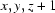; (iv) 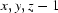; (v) 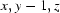.

**Fig. 1.**
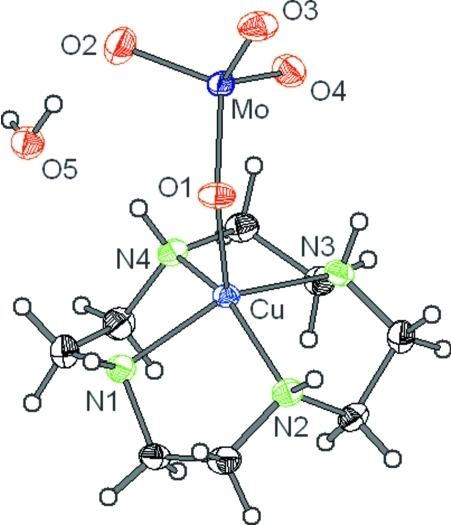
Molecular structure of the [(cyclen)CuMoO4] unit. Thermal ellipsoids are drawn at the 50% probability level.

**Fig. 2.**
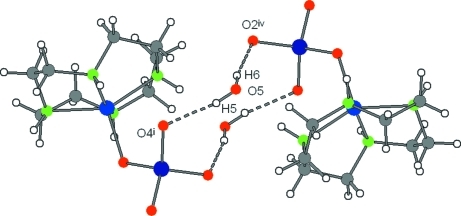
[(cyclen)CuMoO4]2(H2O)2 dimers formed by hydrogen bonds. Symmetry codes: (i) 1 - x, 1 - y, 1 - z; (iv) x, y, -1 + z

**Fig. 3.**
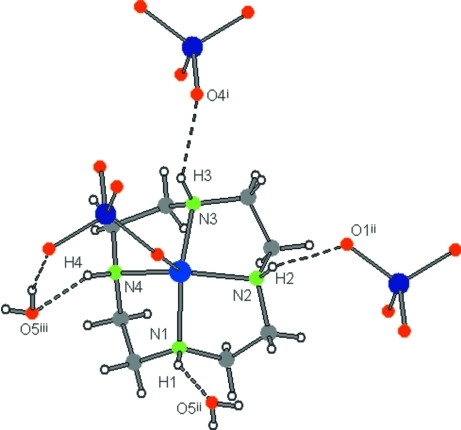
N—H···O hydrogen bonds around the [(cyclen)CuMoO4] unit. Symmetry codes (i) 1 - x, 1 - y, 1 - z; (ii) 2 - x, 1 - y, 1 - z; (iii) x, y, 1 + z

**Fig. 4.**
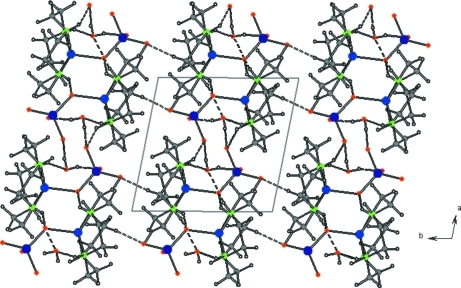
Stacking of the [(cyclen)CuMoO4]H2O layers along the crystallographic b axis.

## supplementary crystallographic information

### Comment

In the title compound (I) the central copper atom is coordinated by four nitrogen atoms of the cyclen ligand and one oxygen atom of the molydate group. This leads to a distorted square pyramidal coordination environment with the four N atoms in the basal positions and the molybdate oxygen atom at the apex. The arrangement of the four nitrogen atoms is nearly co-planar within a maximum deviation of 0.015 Å and the copper atom lies 0.569 Å above this plane. The Cu—N distances ranging from 2.033 (2) to 2.049 (2) Å which is comparable to the Cu—N bond lengths oberserved in other cyclen copper complexes, like [(cyclen)Cu(NO_3_)]NO_3_ (Clay *et al., *1979), [(cyclen)Cu(SCN)]_2_[Ca(NCS)_6_] H_2_O (Lu *et al.*, 1997), [(cyclen)Cu(Ag(CN)_2_)][Ag(CN)_2_] (Yeung *et al.*, 2000) and [(cyclen)Cu(MnN(CN)_5_)] (Guo *et al.*, 2008). The molybdate unit coordinates as a monodentate ligand with a Cu—O distance of 2.104 (2) Å. The Mo—O distances ranging from 1.749 to 1.765 Å for the terminal oxygen atoms. In the case of the µ-bridging O atom O1 the Mo—O distance is slightly enlarged to 1.796 (2) Å. A similar monodentate coordination of molybdate has been observed in the copper complex [LCuMoO_4_]_2_ 5H_2_O (*L* = 1,3,10, 12,16,19-hexaazatetracyclo[17,3,1,1^12.16^,0^4.9^]tetracosane with Cu—O distances of 2.320 (12) and 2.418 (11) Å and Mo—O distances ranging from 1.621 to 1.849 Å (Choi *et al.*, 2004).

Compound (I) contains a water molecule which is involved into two strong hydrogen bonds to the molybdate O2 and O4 oxygen atoms . This leads to [(cyclen)CuMoO_4_]_2_(H_2_O)_2_ dimers containing a centrosymmetric Mo_2_O_6_H_4_ ring of the type *R*_4_^4^(12) (Fig. 2) (Bernstein *et al.*, 1995). Additionally the NH groups of the cyclen ligand are also involved in hydrogen bonds either to water molecules (N1H1 and N4H4) or to molybdate groups (N2H2 and N3H3). As a resuslt of these hydrogen bonds [(cyclen)CuMoO_4_] units and water are interlinked to layers which are oriented parallel to the crystallographic a-c plane. The stacking of the layers in direction of the crystallographic *b* axis is supported by weak C—H···O hydrogen bonds (C···O 3.35 Å) between cyclen ligands and molydate units.

### Experimental

To a stirred suspension of 0.440 mg (2.0 mmol) CuMoO_4_ in 70 ml of aqueous methanol (90%) 0.350 mg (2.0 mmol) cyclen was added. After four hours the blue suspension was filtered and the filtrate evaporated to dryness.Yield. 420 mg (50%). The residue was dissolved in 3 ml of methanol and then the solution was layered with 2-propanol. After one week single crystals of (I) were formed at the methanol/2-propanol interface. IR(cm^-1^): 3172(*s*), 2960(w), 2937(w), 2920(w), 2869(w), 1631(w), 1592(w), 824(*s*); Elemental Analysis [Cu(cyclen)MoO_4_] H_2_O (413.78), C 23.0 (calc. 23.2), H 5.4 (5.4), N 13.1 (13.5) %.

### Refinement

C-bound H atoms of the cyclen ligand were positioned geometrically and refinded using a riding model with U_iso_(H) = 1.2 U_eq_(C) [(C-H) = 0.99 Å]. H atoms attached to N and O were located from difference fourier maps and refined with N-H distances fixed in a range of 0.87 (2) to 0.89 (2) Å and O-H distances fixed at 0.83 (2) and 0.84 (2) Å. The corresponding U_iso_(H) were refined freely.

### Crystal data

**Table:**

[CuMoO_4_(C_8_H_20_N_4_)]·H_2_O	*Z* = 2
*M**_r_* = 413.78	*F*(000) = 418
Triclinic, *P*1	*D*_x_ = 1.990 Mg m^−^^3^
Hall symbol: -P 1	Mo *K*α radiation, λ = 0.71073 Å
*a* = 8.6985 (6) Å	Cell parameters from 16326 reflections
*b* = 8.9784 (6) Å	θ = 3.0–29.6°
*c* = 9.0055 (6) Å	µ = 2.47 mm^−^^1^
α = 90.358 (6)°	*T* = 200 K
β = 91.949 (6)°	Plate, blue
γ = 100.742 (5)°	0.22 × 0.21 × 0.13 mm
*V* = 690.54 (8) Å^3^	

### Data collection

**Table:**

Stoe IPDS 2T diffractometer	3017 independent reflections
Radiation source: fine-focus sealed tube	2788 reflections with *I* > 2σ(*I*)
graphite	*R*_int_ = 0.037
Detector resolution: 6.67 pixels mm^-1^	θ_max_ = 27.0°, θ_min_ = 3.0°
rotation method scans	*h* = −11→11
Absorption correction: numerical (*X-RED*; Stoe & Cie, 2009)	*k* = −11→11
*T*_min_ = 0.612, *T*_max_ = 0.737	*l* = −11→10
9700 measured reflections	

### Refinement

**Table:**

Refinement on *F*^2^	Primary atom site location: structure-invariant direct methods
Least-squares matrix: full	Secondary atom site location: difference Fourier map
*R*[*F*^2^ > 2σ(*F*^2^)] = 0.024	Hydrogen site location: inferred from neighbouring sites
*wR*(*F*^2^) = 0.063	H atoms treated by a mixture of independent and constrained refinement
*S* = 1.04	*w* = 1/[σ^2^(*F*_o_^2^) + (0.0403*P*)^2^ + 0.2037*P*] where *P* = (*F*_o_^2^ + 2*F*_c_^2^)/3
3017 reflections	(Δ/σ)_max_ = 0.002
196 parameters	Δρ_max_ = 0.48 e Å^−^^3^
6 restraints	Δρ_min_ = −1.05 e Å^−^^3^
0 constraints	

### Special details

**Table:**

Geometry. All e.s.d.'s (except the e.s.d. in the dihedral angle between two l.s. planes) are estimated using the full covariance matrix. The cell e.s.d.'s are taken into account individually in the estimation of e.s.d.'s in distances, angles and torsion angles; correlations between e.s.d.'s in cell parameters are only used when they are defined by crystal symmetry. An approximate (isotropic) treatment of cell e.s.d.'s is used for estimating e.s.d.'s involving l.s. planes.
Refinement. Refinement of *F*^2^ against ALL reflections. The weighted *R*-factor *wR* and goodness of fit *S* are based on *F*^2^, conventional *R*-factors *R* are based on *F*, with *F* set to zero for negative *F*^2^. The threshold expression of *F*^2^ > σ(*F*^2^) is used only for calculating *R*-factors(gt) *etc*. and is not relevant to the choice of reflections for refinement. *R*-factors based on *F*^2^ are statistically about twice as large as those based on *F*, and *R*- factors based on ALL data will be even larger.

### Fractional atomic coordinates and isotropic or equivalent isotropic displacement parameters (Å^2^)

**Table:**

	*x*	*y*	*z*	*U*_iso_*/*U*_eq_	
C1	1.0572 (3)	0.1738 (3)	0.7826 (3)	0.0250 (4)	
H1B	0.9796	0.0794	0.7636	0.030*	
H1A	1.1463	0.1485	0.8422	0.030*	
C2	1.1135 (3)	0.2435 (3)	0.6371 (3)	0.0262 (5)	
H2B	1.2008	0.3300	0.6565	0.031*	
H2A	1.1523	0.1675	0.5757	0.031*	
C3	0.8796 (3)	0.1769 (2)	0.4653 (2)	0.0226 (4)	
H3B	0.8488	0.0845	0.5248	0.027*	
H3A	0.9349	0.1504	0.3775	0.027*	
C4	0.7365 (3)	0.2401 (3)	0.4169 (3)	0.0264 (5)	
H4B	0.7672	0.3273	0.3506	0.032*	
H4A	0.6610	0.1614	0.3615	0.032*	
C5	0.5518 (3)	0.1652 (2)	0.6197 (3)	0.0227 (4)	
H5B	0.5989	0.0732	0.6275	0.027*	
H5A	0.4539	0.1403	0.5579	0.027*	
C6	0.5167 (3)	0.2183 (3)	0.7734 (3)	0.0246 (5)	
H6B	0.4571	0.3019	0.7645	0.030*	
H6A	0.4521	0.1338	0.8261	0.030*	
C7	0.7290 (3)	0.1527 (3)	0.9414 (3)	0.0272 (5)	
H7B	0.7284	0.0635	0.8761	0.033*	
H7A	0.6640	0.1198	1.0277	0.033*	
C8	0.8955 (3)	0.2202 (3)	0.9938 (3)	0.0289 (5)	
H8B	0.8945	0.3004	1.0695	0.035*	
H8A	0.9460	0.1405	1.0393	0.035*	
N1	0.9846 (2)	0.2852 (2)	0.8642 (2)	0.0224 (4)	
H1	1.060 (3)	0.356 (3)	0.895 (3)	0.027 (7)*	
N2	0.9827 (2)	0.2966 (2)	0.5561 (2)	0.0214 (4)	
H2	1.023 (3)	0.370 (3)	0.494 (3)	0.028 (7)*	
N3	0.6625 (2)	0.2895 (2)	0.5510 (2)	0.0216 (4)	
H3	0.616 (3)	0.365 (3)	0.526 (4)	0.036 (8)*	
N4	0.6657 (2)	0.2715 (2)	0.8587 (2)	0.0218 (4)	
H4	0.650 (4)	0.339 (3)	0.924 (3)	0.032 (8)*	
O1	0.86208 (19)	0.58802 (17)	0.7174 (2)	0.0254 (3)	
O2	0.6912 (2)	0.7196 (2)	0.9423 (2)	0.0353 (4)	
O3	0.7520 (2)	0.87043 (18)	0.6678 (2)	0.0310 (4)	
O4	0.5312 (2)	0.58763 (19)	0.6764 (2)	0.0290 (4)	
Cu	0.83378 (3)	0.35012 (3)	0.71091 (3)	0.01771 (8)	
Mo	0.70937 (2)	0.692556 (18)	0.750742 (19)	0.01796 (7)	
O5	0.6967 (2)	0.4797 (2)	0.1142 (2)	0.0292 (4)	
H5	0.621 (3)	0.452 (3)	0.168 (3)	0.033 (8)*	
H6	0.678 (5)	0.556 (3)	0.069 (4)	0.058 (12)*	

### Atomic displacement parameters (Å^2^)

**Table:**

	*U*^11^	*U*^22^	*U*^33^	*U*^12^	*U*^13^	*U*^23^
C1	0.0261 (11)	0.0227 (10)	0.0282 (11)	0.0109 (9)	−0.0037 (9)	−0.0040 (9)
C2	0.0220 (11)	0.0245 (11)	0.0330 (12)	0.0070 (9)	0.0008 (9)	−0.0045 (9)
C3	0.0269 (11)	0.0193 (10)	0.0219 (10)	0.0044 (8)	0.0027 (8)	−0.0037 (8)
C4	0.0320 (12)	0.0280 (11)	0.0195 (10)	0.0068 (9)	−0.0017 (9)	−0.0009 (8)
C5	0.0204 (10)	0.0188 (10)	0.0281 (11)	0.0021 (8)	−0.0020 (9)	−0.0031 (8)
C6	0.0189 (10)	0.0226 (10)	0.0319 (12)	0.0029 (8)	0.0021 (9)	−0.0027 (9)
C7	0.0331 (13)	0.0239 (11)	0.0247 (11)	0.0047 (9)	0.0038 (9)	0.0039 (9)
C8	0.0357 (13)	0.0306 (12)	0.0215 (11)	0.0099 (10)	−0.0027 (9)	−0.0001 (9)
N1	0.0219 (9)	0.0202 (9)	0.0251 (9)	0.0053 (7)	−0.0039 (7)	−0.0042 (7)
N2	0.0231 (9)	0.0162 (8)	0.0247 (9)	0.0028 (7)	0.0027 (7)	−0.0005 (7)
N3	0.0233 (9)	0.0183 (8)	0.0245 (9)	0.0078 (7)	−0.0026 (7)	0.0005 (7)
N4	0.0255 (9)	0.0164 (8)	0.0238 (9)	0.0048 (7)	0.0005 (7)	−0.0032 (7)
O1	0.0254 (8)	0.0135 (7)	0.0378 (9)	0.0048 (6)	0.0036 (7)	0.0007 (6)
O2	0.0475 (11)	0.0360 (10)	0.0237 (9)	0.0112 (8)	0.0024 (8)	−0.0024 (7)
O3	0.0388 (10)	0.0194 (8)	0.0370 (10)	0.0095 (7)	0.0086 (8)	0.0038 (7)
O4	0.0268 (9)	0.0279 (8)	0.0325 (9)	0.0062 (7)	−0.0039 (7)	−0.0001 (7)
Cu	0.01851 (14)	0.01362 (13)	0.02126 (14)	0.00388 (10)	−0.00018 (10)	−0.00115 (10)
Mo	0.02191 (11)	0.01353 (10)	0.01901 (11)	0.00478 (7)	0.00084 (7)	0.00004 (7)
O5	0.0301 (9)	0.0259 (8)	0.0324 (9)	0.0067 (7)	0.0048 (7)	−0.0017 (7)

### Geometric parameters (Å, °)

**Table:**

C1—N1	1.482 (3)	C7—C8	1.520 (4)
C1—C2	1.513 (3)	C7—H7B	0.9900
C1—H1B	0.9900	C7—H7A	0.9900
C1—H1A	0.9900	C8—N1	1.484 (3)
C2—N2	1.484 (3)	C8—H8B	0.9900
C2—H2B	0.9900	C8—H8A	0.9900
C2—H2A	0.9900	N1—Cu	2.034 (2)
C3—N2	1.486 (3)	N1—H1	0.864 (17)
C3—C4	1.513 (3)	N2—Cu	2.0493 (19)
C3—H3B	0.9900	N2—H2	0.890 (17)
C3—H3A	0.9900	N3—Cu	2.033 (2)
C4—N3	1.491 (3)	N3—H3	0.881 (18)
C4—H4B	0.9900	N4—Cu	2.0413 (19)
C4—H4A	0.9900	N4—H4	0.875 (17)
C5—N3	1.485 (3)	O1—Mo	1.7955 (16)
C5—C6	1.520 (3)	O1—Cu	2.1043 (15)
C5—H5B	0.9900	O2—Mo	1.7571 (18)
C5—H5A	0.9900	O3—Mo	1.7490 (16)
C6—N4	1.482 (3)	O4—Mo	1.7652 (17)
C6—H6B	0.9900	O5—H5	0.829 (18)
C6—H6A	0.9900	O5—H6	0.838 (19)
C7—N4	1.482 (3)		
			
N1—C1—C2	108.15 (18)	N1—C8—H8A	109.9
N1—C1—H1B	110.1	C7—C8—H8A	109.9
C2—C1—H1B	110.1	H8B—C8—H8A	108.3
N1—C1—H1A	110.1	C1—N1—C8	113.52 (18)
C2—C1—H1A	110.1	C1—N1—Cu	103.80 (14)
H1B—C1—H1A	108.4	C8—N1—Cu	109.08 (15)
N2—C2—C1	109.64 (18)	C1—N1—H1	107 (2)
N2—C2—H2B	109.7	C8—N1—H1	109 (2)
C1—C2—H2B	109.7	Cu—N1—H1	115 (2)
N2—C2—H2A	109.7	C2—N2—C3	114.06 (17)
C1—C2—H2A	109.7	C2—N2—Cu	107.64 (14)
H2B—C2—H2A	108.2	C3—N2—Cu	102.43 (13)
N2—C3—C4	107.08 (18)	C2—N2—H2	108.6 (19)
N2—C3—H3B	110.3	C3—N2—H2	107.2 (19)
C4—C3—H3B	110.3	Cu—N2—H2	117 (2)
N2—C3—H3A	110.3	C5—N3—C4	113.35 (17)
C4—C3—H3A	110.3	C5—N3—Cu	103.78 (14)
H3B—C3—H3A	108.6	C4—N3—Cu	107.78 (14)
N3—C4—C3	109.07 (18)	C5—N3—H3	111 (2)
N3—C4—H4B	109.9	C4—N3—H3	109 (2)
C3—C4—H4B	109.9	Cu—N3—H3	112 (2)
N3—C4—H4A	109.9	C6—N4—C7	115.36 (18)
C3—C4—H4A	109.9	C6—N4—Cu	107.91 (14)
H4B—C4—H4A	108.3	C7—N4—Cu	104.25 (14)
N3—C5—C6	108.09 (17)	C6—N4—H4	108 (2)
N3—C5—H5B	110.1	C7—N4—H4	107 (2)
C6—C5—H5B	110.1	Cu—N4—H4	114.3 (19)
N3—C5—H5A	110.1	Mo—O1—Cu	125.18 (8)
C6—C5—H5A	110.1	N3—Cu—N1	148.36 (7)
H5B—C5—H5A	108.4	N3—Cu—N4	85.93 (8)
N4—C6—C5	109.38 (18)	N1—Cu—N4	84.96 (8)
N4—C6—H6B	109.8	N3—Cu—N2	85.57 (8)
C5—C6—H6B	109.8	N1—Cu—N2	85.70 (8)
N4—C6—H6A	109.8	N4—Cu—N2	146.84 (7)
C5—C6—H6A	109.8	N3—Cu—O1	102.97 (7)
H6B—C6—H6A	108.2	N1—Cu—O1	108.66 (7)
N4—C7—C8	107.65 (18)	N4—Cu—O1	106.23 (7)
N4—C7—H7B	110.2	N2—Cu—O1	106.91 (7)
C8—C7—H7B	110.2	O3—Mo—O2	108.43 (9)
N4—C7—H7A	110.2	O3—Mo—O4	110.51 (9)
C8—C7—H7A	110.2	O2—Mo—O4	108.75 (9)
H7B—C7—H7A	108.5	O3—Mo—O1	110.04 (8)
N1—C8—C7	108.83 (19)	O2—Mo—O1	110.65 (9)
N1—C8—H8B	109.9	O4—Mo—O1	108.45 (8)
C7—C8—H8B	109.9	H5—O5—H6	106 (4)
			
N1—C1—C2—N2	−53.5 (2)	C1—N1—Cu—N4	121.02 (14)
N2—C3—C4—N3	−55.8 (2)	C8—N1—Cu—N4	−0.30 (15)
N3—C5—C6—N4	−53.4 (2)	C1—N1—Cu—N2	−27.13 (14)
N4—C7—C8—N1	−53.0 (2)	C8—N1—Cu—N2	−148.44 (15)
C2—C1—N1—C8	168.05 (19)	C1—N1—Cu—O1	−133.53 (13)
C2—C1—N1—Cu	49.76 (19)	C8—N1—Cu—O1	105.15 (15)
C7—C8—N1—C1	−87.1 (2)	C6—N4—Cu—N3	−1.03 (14)
C7—C8—N1—Cu	28.1 (2)	C7—N4—Cu—N3	122.10 (15)
C1—C2—N2—C3	−84.7 (2)	C6—N4—Cu—N1	−150.70 (15)
C1—C2—N2—Cu	28.2 (2)	C7—N4—Cu—N1	−27.57 (14)
C4—C3—N2—C2	168.62 (18)	C6—N4—Cu—N2	−76.5 (2)
C4—C3—N2—Cu	52.62 (18)	C7—N4—Cu—N2	46.6 (2)
C6—C5—N3—C4	165.92 (19)	C6—N4—Cu—O1	101.31 (14)
C6—C5—N3—Cu	49.29 (19)	C7—N4—Cu—O1	−135.56 (14)
C3—C4—N3—C5	−85.7 (2)	C2—N2—Cu—N3	−150.00 (14)
C3—C4—N3—Cu	28.5 (2)	C3—N2—Cu—N3	−29.46 (13)
C5—C6—N4—C7	−87.4 (2)	C2—N2—Cu—N1	−0.44 (13)
C5—C6—N4—Cu	28.7 (2)	C3—N2—Cu—N1	120.10 (14)
C8—C7—N4—C6	168.22 (19)	C2—N2—Cu—N4	−74.40 (19)
C8—C7—N4—Cu	50.08 (19)	C3—N2—Cu—N4	46.1 (2)
C5—N3—Cu—N1	46.9 (2)	C2—N2—Cu—O1	107.77 (13)
C4—N3—Cu—N1	−73.6 (2)	C3—N2—Cu—O1	−131.69 (13)
C5—N3—Cu—N4	−26.66 (13)	Mo—O1—Cu—N3	56.76 (13)
C4—N3—Cu—N4	−147.15 (14)	Mo—O1—Cu—N1	−122.84 (11)
C5—N3—Cu—N2	121.25 (14)	Mo—O1—Cu—N4	−32.74 (13)
C4—N3—Cu—N2	0.77 (14)	Mo—O1—Cu—N2	146.02 (11)
C5—N3—Cu—O1	−132.40 (13)	Cu—O1—Mo—O3	−153.37 (11)
C4—N3—Cu—O1	107.12 (14)	Cu—O1—Mo—O2	86.82 (12)
C1—N1—Cu—N3	47.2 (2)	Cu—O1—Mo—O4	−32.38 (13)
C8—N1—Cu—N3	−74.1 (2)		

### Hydrogen-bond geometry (Å, °)

**Table:**

*D*—H···*A*	*D*—H	H···*A*	*D*···*A*	*D*—H···*A*
O5—H5···O4^i^	0.83 (2)	1.95 (2)	2.770 (2)	169 (3)
N1—H1···O5^ii^	0.86 (2)	2.35 (2)	3.156 (3)	156 (3)
N2—H2···O1^ii^	0.89 (2)	2.19 (2)	2.949 (2)	143 (3)
N3—H3···O4^i^	0.88 (2)	2.28 (3)	2.955 (3)	133 (3)
N4—H4···O5^iii^	0.88 (2)	2.10 (2)	2.928 (3)	157 (3)
O5—H6···O2^iv^	0.84 (2)	1.85 (2)	2.666 (3)	163 (4)
C3—H3B···O3^v^	0.99	2.36	3.345 (3)	175

Symmetry codes: (i) −*x*+1, −*y*+1, −*z*+1; (ii) −*x*+2, −*y*+1, −*z*+1; (iii) *x*, *y*, *z*+1; (iv) *x*, *y*, *z*−1; (v) *x*, *y*−1, *z*.
